# Exploring physicians and patients’ perspectives for current interventions on thyroid nodules using a MCDA method

**DOI:** 10.1186/s12962-021-00279-3

**Published:** 2021-05-01

**Authors:** Linda Karrer, Shixuan Zhang, Thomas Kühlein, Peter L. Kolominsky-Rabas

**Affiliations:** 1 Interdisciplinary Centre for Health Technology Assessment (HTA) and Public Health (IZPH), Friedrich-Alexander-University of Erlangen-Nuremberg, Erlange, Bavaria Germany; 2 Institute of General Practice, Friedrich-Alexander-University of Erlangen-Nuremberg, Erlangen, Bavaria Germany; 3 National Leading-Edge Cluster Medical Technologies “Medical Valley EMN”, Erlangen, Bavaria Germany

**Keywords:** Thyroid nodules, Multi criteria decision Analysis, MCDA, EVIDEM, Overdiagnosis, Adverse Cascade Effects, Health Technology Assessment (HTA)

## Abstract

**Background:**

The detection of thyroid cancer has rapidly increased over last few decades without an increase in disease specific mortality. Several studies claim that the diagnose of thyroid nodules through routine ultrasound imaging is often the trigger for cascade effects leading to unnecessary follow-up over many years or to invasive treatment. The objective of this study was to explore physicians’ and patients’ insights and preferences regarding the current interventions on thyroid nodules.

**Methods:**

An online survey was developed using a comprehensive multi-criteria decision analysis (MCDA) framework, the EVIdence based Decision-Making (EVIDEM). The EVIDEM core model used in this study encompassed 13 quantitative criteria and four qualitative criteria. Participants were asked to provide weights referring to what matters most important in general for each criterion, performance scores for appraising the interventions on thyroid nodules and their consideration of impact of contextual criteria. Normalized weights and standardized scores were combined to calculate a value contribution across all participants, additionally differences across physicians and patients’ group were explored.

**Results:**

48 patients and 31 physicians were included in the analysis. The value estimate of the interventions on thyroid nodules reached 0.549 for patients’ group and 0.5 was reported by the physicians’ group, compared to 0.543 for all participants. The highest value contributor was ‘Comparative effectiveness’ (0.073 ± 0.020). For the physicians’ group, ‘Comparative safety’ (0.050 ± 0.023) was given with higher value. And for the patients’ group, ‘Type of preventive benefits’ (0.059 ± 0.022) contributed more positively to the value estimation. 51% participants considered ‘Population priorities and access’ having a negative impact on the interventions of nodules.66% participants thought that the ‘system capacity’ had a negative impact.

**Conclusion:**

Our study shows participants’ preferences on each criterion, i.e., physician indicated keeping the interventions safe and effective more important, patients indicated quality of life after receiving interventions more important. Through comparison among participants, differences have been highlighted, which can make better communication between physicians and patients. This study provides a supportive decision-making for healthcare providers when they explored the interventions on thyroid nodules.

## Background

Thyroid nodule (referred to below also as nodule) refers to an abnormal growth of thyroid cells that form a lump within the thyroid gland [1]. They are very common, according to the American Thyroid Association (ATA), about 50% of all people by age 60 have nodules [1]. Ultrasonography can detect nodules of any size in up to 67% of the general population [2]. Over 90% of nodules are benign, harmless and noncancerous [1], but still 5–10% of nodules being malignant as thyroid cancer [3]. In order to detect thyroid cancer, most nodules need further evaluation, such as control thyroid ultrasonography over time, thyroid hormone test, scintigraphy or fine needle aspiration cytology. In addition, the decision to conduct thyroid surgery is made on therapeutic or diagnostic grounds [3, 4].

In 2012, about 230,000 new cases of thyroid cancer were estimated among women and 70,000 among men, with an age-standardized rate of 6.10/100,000 women und 1.90/100.000 men [5]. The detection and thus the incidence of thyroid cancer has rapidly increased over the last few decades without an increase in disease specific mortality [6]. The largest increase has been observed in South Korea, the rate of thyroid cancer diagnoses in 2011 (about 69/100,000) was 15 times that observed in 1993 (about 4/100,000), but with a stable mortality [7, 8]. According to a survey of cancer incidence (1960–2007) in Five Continents conducted by the International Agency for Research on Cancer, the incidence of thyroid cancer steady raised in many countries in contrast to the declines in mortality [9]. Among them, the incidence more than doubled has been seen in France, Italy, Croatia, the Czech Republic, Israel, China, Australia, Canada and the United States [9]. Several studies from most industrialized countries have shown the increasing numbers of thyroid cancer with constant or decreasing mortality [6–9].

Significant increase in thyroid cancer in developed countries is attributed mainly to an unapproved diagnostic imaging of the thyroid gland by ultrasound [7, 10, 11]. This estimation can be seen also in different incidence of thyroid nodules under different imaging. Ultrasound offers the highest sensitivity and detects incidental thyroid nodules in 40%-67% of patients [12–15]. Incidental thyroid nodules are less prevalent on Computed tomography (CT), about 16%-25%. [16–19]. Palpable thyroid nodules occur only in 4%-7% of the population [20].

The diagnose of nodules through routine ultrasound imaging is often the trigger for cascade effects leading to unnecessary follow-up over many years or to invasive treatment, which has been claimed as overdiagnosis and overtreatment [21]. The diagnosis of nodules leads to a growing amount of thyroid surgery [22]. According to an international comparison study in 2015, the rate of thyroid surgery in Germany (109/100,000/year) was about 2.5 times higher than that in the USA, four times higher than that in England and seven times higher than the rate of Netherland [23]. Although the rate in other countries in contrast to that in Germany was declining, but the number was still elevated [23]. Many thyroid surgeries after which histology reveals benign nodules that would not have needed to be removed [22]. Thyroid surgery to whom will never be symptomatic is not only costly to the individual and the healthcare system, but also can bring lifelong effects [6, 7]. Most must receive lifelong thyroid-replacement therapy, a few have complications from the surgery procedure. An analysis of South Korea’s insurance system claims for more than 15,000 Korean who underwent surgery showed that 11% had hypoparathyroidism and 2% had vocal-cord paralysis [7].

Several studies have addressed the overdiagnosis and overtreatment of thyroid cancer. The major reason of overdiagnosis and overtreatment of thyroid cancer is, in order to prevent 5–10% of nodules being malignant as thyroid cancer, further evaluation and treatment have been done to patients with thyroid nodules. Little is known about the perspectives and preferences of physicians and patients regarding the current interventions on thyroid nodules. The objective of this study is to explore physicians and patients’ insights and preferences for current interventions on thyroid nodules. using a multi-criteria decision analysis (MCDA) method.

## Methods

### Study design

MCDA is an umbrella term to describe a collection of formal approaches which seek to take explicit account of multiple criteria in helping individuals or groups explore decision that matter [24]. Since shared decision making between physicians and patients becomes a core concept of patient-centered care system, taking the preferences, insights from both physicians and patients can reduce decision conflicts and get an overview of all stakeholders related to thyroid nodules [25]. MCDA can provide a transparent and structured process to help us to achieve this objective.

The EVIdence based decision-making (EVIDEM, 10th Edition 2019) framework as an open source of MCDA tool was selected to investigate participants’ insights and preferences. EVIDEM framework is designed to reflect and to stimulate structured reflection and pragmatic collection of preferences on healthcare interventions from all participants, through a broad spectrum of quantitative and qualitative criteria [26]. In this study, we provided synthesized data for each of these criteria to create a specific EVIDEM core model online questionnaire regarding the interventions on thyroid nodules. The synthesized data we provided are based on discussion of expert groups, the constructed questionnaire has been done through two rounds inner validation.

### Study participants

Participants were recruited through public access like medical networks, regional distributors and newspaper announcement. All participants were recruited anonymously and voluntarily. The online survey was planned to launch from 2018 November 20th to 2019 June 30th. At least 25 physicians and 25 patients were planned to include in the data analysis. Inclusion criteria of participants for further analysis were: physicians; patients who ever had thyroid disease. In contrast, normal citizens who had no thyroid disease before will be excluded for further analysis. All participants were invited to fill in an online questionnaire. The online questionnaire was constructed through the survey software EFS Survey by UNIPARK (https://www.unipark.com/umfragesoftware/) [27]. Informed written consent and online questionnaire were approved by „Data protection officer“ at FAU according to the Bavarian State Representative Data Protection (https://www.datenschutz-bayern.de/vorstell/impressum.html). We conducted an online survey open to the public, informed written consent was clicked while fulfilling the online survey by each participant. Participants were assured that the research would not contain their personal identifying information.

### Online questionnaire design and conduct

An online survey was created in line with the core model of EVIDEM framework for the participants. The EVIDEM core model used in this study encompassed five categories in total 13 quantitative criteria, while the contextual tool consisted of four qualitative criteria. The description and example question of each category and criterion used in this study is shown in Table 1. A definition of all criteria as well as background knowledge such as sociodemographic data used in this survey was provided to participants in the online questionnaire in German language. To provide sufficient evidence to appraise each criterion, a literature review was used to obtain relevant information on thyroid nodule and its current management [8, 28].

**Table 1:** MCDA EVIDEM Framework 10th Edition adapted to evaluation of interventions on thyroid nodules

Quantitative criteria: MCDA core model		Weighting example question	Scoring example question
Category	Criterion	How important do you consider the following criteria for decisions regarding healthcare interventions in general? (Weighting scale from 1 to 5, 5 is the highest relative importance)	Please evaluate the following criteria in connection with the current interventions on thyroid nodules (For non-comparative criteria from 0 to + 5, for comparative criteria from -5 to + 5, 5 is the highest score)
Need for intervention	Disease severity	How important is disease severity for the need of interventions?	How severe is the disease as a thyroid nodule?
	Size of affected population	How important is the size of the population affected for the need of interventions?	How big is the affected population?
	Unmet needs	How important is unmet need (that requirement cannot be met with current treatments) for the need of interventions?	Is there a large unmet need (i.e. need cannot be met with current treatments) for the interventions on thyroid nodules?
Comparative outcomes of intervention	Comparative efficacy/effectiveness	How important is effectiveness of the intervention?	How effective is the interventions on thyroid nodules?
	Comparative safety/tolerability	How important is safety of the intervention?	How safe is the interventions on thyroid nodules?
	Comparative patient-perceived health	How important are patient-relevant endpoints after the intervention, e.g. health-related quality of life?	How should the interventions on thyroid nodules be assessed with regard to patient-relevant endpoints (e.g. quality of life)?
Type of benefit provided by intervention	Type of preventive benefit	How important are preventive benefits, e.g. reducing disease transmission?	How is the preventive benefit of the current interventions on thyroid nodules?
	Type of therapeutic benefit	How important are therapeutic benefits, e.g. healing, symptom relief?	How is the therapeutic benefit of the current interventions on thyroid nodules?
Comparative economic consequences of intervention	Comparative cost of intervention	How important are economic impacts as intervention costs to be considered?	How high are the costs of interventions on thyroid nodule?
	Comparative other medical costs	How important are economic impacts as other medical costs (for example, by through side effects, hospital stays) to be considered?	How high are the costs for other medical costs due to a thyroid nodule?
	Comparative non-medical costs	How important are economic impacts as non-medical costs (for example, by disability, care) to be considered?	How high are the costs for non-medical costs due to a thyroid nodule?
Knowledge about the intervention	Quality of evidence	How important is the quality of scientific studies about the interventions?	How is the quality of scientific studies about the interventions on thyroid diseases?
	Clinical practice guidelines	How important is in-depth knowledge of expert opinions and guidelines about interventions?	How do you assess the interventions on thyroid nodules recommended in recognized guidelines?
Contextual Criteria: MCDA Contextual Tool		Please assess how the following criteria impact the current interventions on thyroid nodules (negative impact: -1, neutral impact: 0, positive impact: 1)	
Mandate and scope of the healthcare system		How are the healthcare system influence of the interventions on thyroid nodules?	
System capacity and appropriate use of intervention		How do you assess the system capacity and appropriate use of the interventions on thyroid nodules?	
Population priorities and access		How are population priorities and access to intervention influence of the interventions on thyroid nodules?	
Political/historical/cultural context		What are the effects of political, historical and / or cultural circumstances on the interventions on thyroid nodules?	

In the first part of the survey, participants’ perspectives on what matters most important in general, i.e., which criterion contributes the most to the value of healthcare interventions, was captured by weight. Our study used a 5-point weighting scale (1 = lowest relative importance, 5 = highest relative importance). In the second part of the survey, participants were asked to appraise the actual intervention on thyroid nodules about its performance for each criterion, which captured by score. Participants scored performance of the intervention on thyroid nodules using two types of scoring scale, for non-comparative criteria from 0 to + 5, for comparative criteria from -5 to + 5. Higher score indicates better performance. The third part of the survey was about qualitative contextual criteria, participants indicates whether consideration of a given criterion had a negative, neutral, or positive impact on the decision about the interventions on thyroid nodules. This part of survey includes qualitative contextual criteria, which has not been accounted to the quantitative result, just a support tool helping researchers to understand how the given criteria impact their decision making. A numerical scale (-1, 0, 1) was used to represent negative impact, neutral impact and positive impact.

### Data analysis

Numerical outputs were calculated for each participant. In this study, we used Excel to calculate and analyze weight, score and contextual impact. Mean and standard deviations (SD) were calculated in Excel to quantify the variability as descriptive statistics. Normalized weights were summed up to 1.0 (Wx, Σ Wx = 1). For example, for one single participant, the normalized weight of each criterion equals to the weight of this criterion given by this participant divided by the total weights given by this participant. For example, participant A weighted a criterion with a “5”, and total weight of all criteria given by participant A was “50”, then the normalized weight for the criterion given by participant A was 5/50 = 0.1. The scores are standardized on a scale of 0 to 1. The value contribution (VCx) of each criterion was calculated following a linear additive model as sum of the products of the normalized weights and standardized scores.. For example, if a criterion received a normalized weight of 0.05, and a standardized score of 1, its value contribution is 0.05*1 = 0.05. For the evaluation of the contextual criteria, a numerical scale (-1, 0, 1) was used to represent negative impact, neutral impact and positive impact. The number and percentage of the choice has been summarized and discussed in the text.

## Results

The online survey was launched from 2018 November 20th to 2019 June 30th. At the point of closing the online system on 2019 June 30th, we had received valid data from 105 participants in total. After application of inclusion and exclusion criteria to achieve study objective, 26 normal citizens who had no thyroid disease were excluded. Data from 31 physicians and 48 patients were included in the analysis. Of all physician participants, 58.1% are from general practice and 19.3% from internal medicine. Of all patient participants, 41.7% have thyroid nodules and 12.5% have been conducted total thyroidectomy. Other sociodemographic data has been listed in Table 2.

**Table 2:** Sociodemographic data of participants

Characteristics	Physicians	Patients
Number of participants	31	48
Age (years)		
< 20	0	0
20–29	1	1
30–39	4	1
40–49	7	6
50–59	8	10
60–69	8	12
> 70	3	18
Medical specialists of Physicians		
General practice	18	58.1%
Internal medicine	6	19.4%
Surgery	3	9.6%
Radiology	1	3.2%
Otolaryngology	1	3.2%
Other fields	2	6.4%
Kinds of thyroid disease of patients		
Hashimoto	12	25%
Nodules	20	41.7%
Hyperfunction	2	4.2%
Subfunction	4	8.3%
Goiter	4	8.3%
Total thyroidectomy	6	12.5%

### Perspectives of participants on decision criteria

**Fig. 1:**
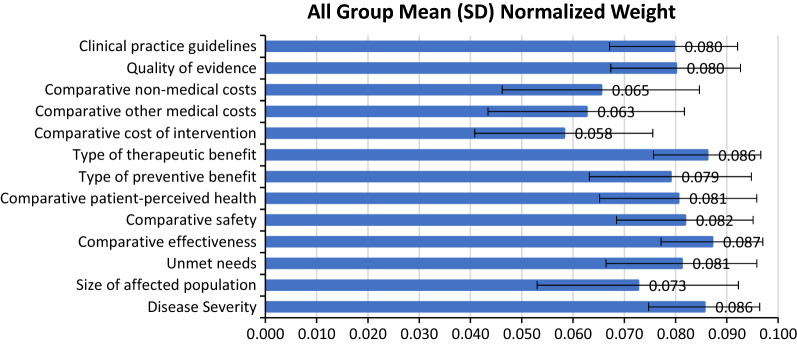
Weights for each criterion of the EVIDEM Core Model for all study participants. A 5-point weighting scale was used (1 = low importance, 5 = high importance). The mean of weights for each criterion and its standard deviation (SD) were normalized to sum up to 1

Normalized weight across criteria was summing up to 1.0, higher weight indicates that the criterion is more important from participants’ view. Regarding weights provided by all participants, Fig. 1 shows that the most important criteria was ‘Comparative effectiveness (0.087 ± 0.010)’, followed by ‘Type of therapeutic benefit (0.086 ± 0.010)’ and ‘Disease severity (0.086 ± 0.011)’. Mean contribution of weights was 0.077. Relative important criteria for all participants were: ‘Comparative safety’, ‘Unmet needs’, ‘Comparative patient-perceived health’, ‘Clinical practice guidelines’, ‘Quality of evidence’, and ‘Type of preventive benefit’. As Fig. 1 shows, the least important criteria were three cost consequences of intervention relative criteria. The largest variations in weights were observed for ‘Size of affected population (SD, 0.020)’, ‘Comparative non-medical costs (SD, 0.019)’and ‘Comparative other medical costs (SD, 0.019)’The smallest variations were ‘Comparative effectiveness (SD, 0.010)’, ‘Type of therapeutic benefit (SD, 0.010)’ and ‘Disease Severity (SD, 0.011)’.

**Fig. 2:**
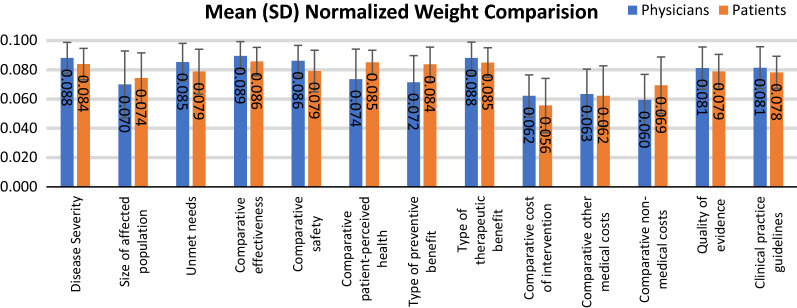
Mean normalized weights assigned to patients’ group and physicians’ group. A 5-point weighting scale was used (1 = low importance, 5 = high importance). The mean of weights for each criterion and its standard deviation (SD) were normalized to sum up to 1

Mean normalized weights were also calculated assigned to each criterion by physicians’ group and patients’ group (Shown in Fig. 2). For physicians’ group, mean weight contribution was 0.077. ‘Comparative effectiveness (0.089 ± 0.010)’ was the most important criterion, followed by ‘Type of therapeutic benefit (0.088 ± 0.011)’ and ‘Disease severity (0.088 ± 0.011)’. For patients’ group, the most important criterion was also ‘Comparative effectiveness (0.086 ± 0.010)’, followed by ‘Type of therapeutic benefit (0.085 ± 0.010)’ and ‘Comparative patient-perceived health (0.085 ± 0.008)’. Physicians weighted ‘Comparative safety (0.086 ± 0.011)’ and ‘Unmet needs (0.085 ± 0.013)’ much higher than patients’ group, for patients’ group were 0.079 ± 0.014 and 0.079 ± 0.015 respectively. Large variance was also showed in ‘Type of preventive benefits’, for patients’ group was 0.084 ± 0.012, however, in the physicians’ group, the weight was much lower than that with 0.072 ± 0.018. Similar situation also happened to the criterion of ‘Comparative patient-perceived health’, the weights given by physicians was 0.074 ± 0.021, lower than that for patients 0.085 ± 0.008.

### Scores

**Fig. 3:**
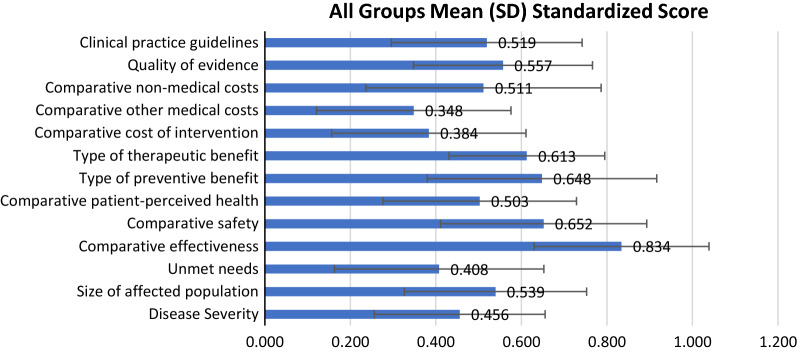
Mean (SD) standardized score for intervention on thyroid nodules assigned to each criterion by all participants. For non-comparative criteria ranging from 0 to + 5, for comparative criteria ranging from − 5 to + 5, these scores were standardized on a scale of 0 to 1

As Fig. 3 shows, for non-comparative criteria, ‘Type of preventive benefit’ received the highest score (0.648 ± 0.268), which shows most participants gave highest performances score on the preventive methods for thyroid nodules. Followed by ‘Type of therapeutic benefit’, this was scored 0.613 ± 0.182. Especially this criterion had the smallest SD, which indicates most participants have an agreement on therapeutic methods are highly useful for the intervention of thyroid nodules. The two criteria ‘Quality of evidence (0.557 ± 0.209)’ and ‘Clinical practice guidelines (0.519 ± 0.223)’ in the category of knowledge of the intervention received also relative higher score from all participants. For the category ‘Need of intervention’, ‘Unmet needs’ received the smallest score 0.408 ± 0.245, followed by ‘Disease Severity’, with a score of 0.456 ± 0.200. But ‘Size of affected population’ in the same category received a higher score 0.539 ± 0.213.

For comparative criteria, ‘Comparative effectiveness’ received the highest score, 0.834 ± 0.204. ‘Comparative patient perceived health’ in the same category ‘Treatment Interventions’ got a lower score of 0.503 ± 0.226. We designed this question as ‘How should the interventions on thyroid nodules be assessed with regard to patient-relevant endpoints (e.g. quality of life)?’ The lowest scores were observed for ‘Comparative cost of intervention’ (0.384 ± 0.227)and ‘Comparative other medical costs’ (0.348 ± 0.228).

**Fig. 4:**
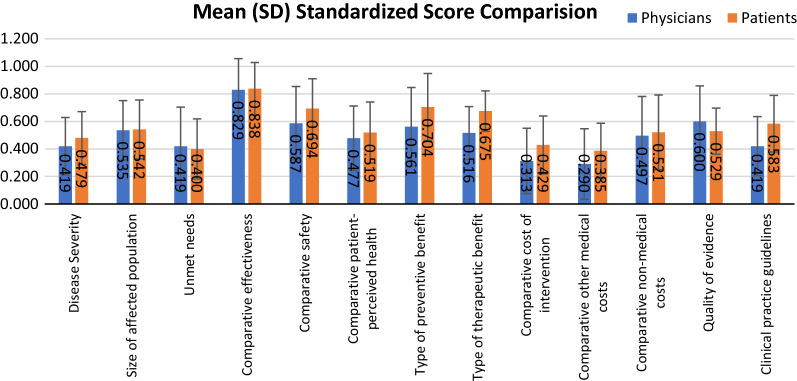
Mean (SD) standardized scores assigned to each criterion by physicians’ group and patients’ group. For non-comparative criteria ranging from 0 to + 5, for comparative criteria ranging from -5 to + 5, these scores were standardized on a scale of 0 to 1

As shown in Fig. 4, we also calculated mean (SD) standardized scores comparison between physicians’ group and patients’ group. Both physicians’ group and patients’ group gave the impact on ‘comparative effectiveness’ the highest score (physicians’ group: 0.829 ± 0.227, patients’ group: 0.838 ± 0.191). Large variance between two groups was observed from four criteria: ‘Type of therapeutic benefit’, ‘Type of preventive benefit’, ‘Comparative safety’ and ‘Clinical practice guidelines’. Referring to ‘Comparative safety’, the score of the physicians’ group (0.587 ± 0.267) was smaller than patients’ group (0.694 ± 0.216).. Another interesting criterion was ‘Clinical practice guidelines’, the score of the physicians’ group (0.419 ± 0.215) was also smaller than that from patients’ group (0.583 ± 0.206). Similar situation also happened with ‘Type of therapeutic benefit’ and ‘Type of preventive benefit’, scores from the patients’ group (0.704 ± 0.244, 0.675 ± 0.147, respectively) were higher than those from physicians’ group (0.516 ± 0.192, 0.561 ± 0.285, respectively).

### Value contribution

**Fig. 5:**
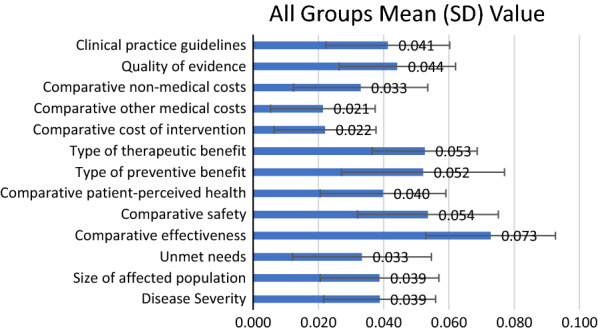
Mean (SD) value contributions for interventions on thyroid nodules assigned to each criterion by all participants

Figure 5 shows the mean (SD) value contribution of interventions on nodules from all participants after adjusting the performance scores to the weights for each criterion. The value estimate of all criteria by all participants was 0.543 on a scale of 0 to 1. The highest value contributor was the ‘Comparative effectiveness’ (0.073 ± 0.020), followed by ‘Comparative safety’ (0.054 ± 0.022), ‘Type of therapeutic benefit’ (0.053 ± 0.016) and ‘Type of preventive benefits’ (0.052 ± 0.025). For comparative criteria, three cost consequences of intervention relative criteria were negatively contributed to the value. For non-comparative criteria, the value contribution of ‘Unmet needs’ (0.033 ± 0.021), ‘Size of affected populations’ (0.039 ± 0.018) and ‘Disease Severity’ (0.039 ± 0.017) were also relative low.

**Fig. 6:**
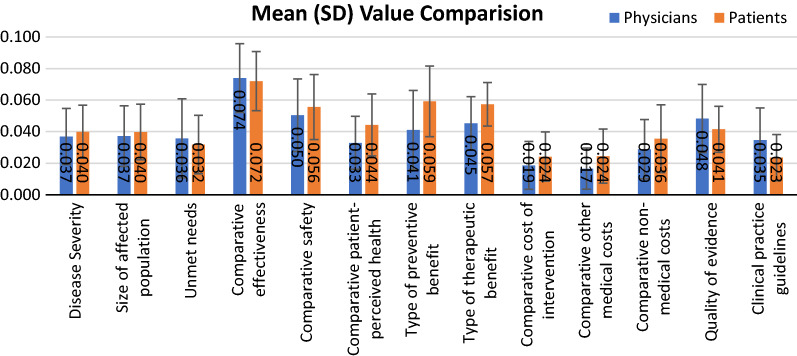
Mean (SD) standardized value contribution assigned to each criterion by different stakeholder groups as physicians’ group and patients’ group

As shown in Fig. 6, the value estimate of the interventions on nodules reached 0.5 for physicians’ group, and 0.549 was observed from patients’ group. This figure shows very clear that different stakeholders’ preferences and thoughts are different. In the physicians’ group, the highest value contributor was the ‘Comparative effectiveness’ (0.074 ± 0.022), followed by the ‘Comparative safety’ (0.050 ± 0.023). In the patients’ group, the highest value contributor was also the ‘Comparative effectiveness’ (0.072 ± 0.019), followed by ‘Type of preventive benefits’ (0.059 ± 0.022).

###  Impacts of contextual criteria

**Fig. 7:**
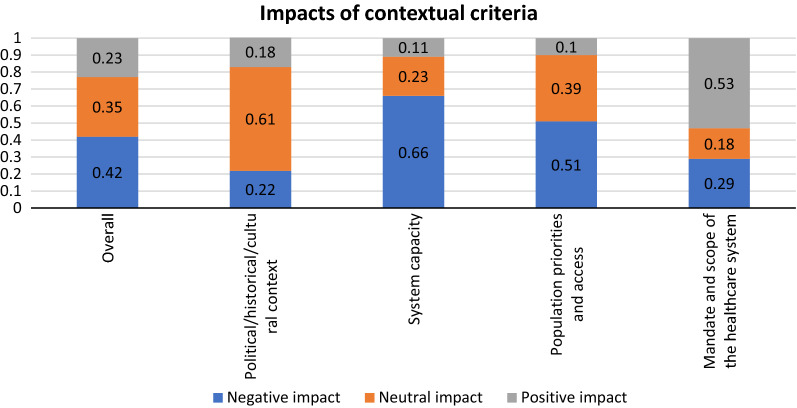
Impacts of contextual criteria on the appraisal of intervention on thyroid nodules by participants, as percentage of impacts (negative, neutral, or positive) assigned for a given criterion

Figure 7 illustrates qualitative contextual criteria. 53% participants considered ‘Mandate and scope of the healthcare system’ (How are the healthcare system influence of the interventions on thyroid nodules?) had a positive impact. Consideration of ‘System capacity and appropriate use of intervention’, 66% participants thought it had a negative impact, and for ‘Population priorities and access’, with the question “How are population priorities and access to intervention influence of the interventions on thyroid nodules?”, also 51% participants considered it had a negative impact. Nearly 61% participants thought ‘Political/historical/cultural context’ had neutral impact on the intervention of thyroid nodules. The overall negative impact, neutral impact and positive impact was 42, 35, and 23%, respectively.

## Discussion

### Using online platform to conduct MCDA

This study refers to the current issues which are discussed in many countries’ healthcare system [8, 28–32]. The incidence of thyroid cancer has been increasing faster than other cancer because of the prevalence of low risk, non-lethal tumors from detection of a large subclinical reservoir of disease [30]. This increased incidence of thyroid cancer with attribution to over-diagnosis has been described in most developed countries where patients have high access to health detection. The study estimated that 70% to 90% of these patients had asymptomatic lesions during lifetime if ultrasound and other imaging studies were not available [30]. Our study used MCDA to identify the perspective and preference of thyroid nodules interventions from physicians’ and patients’ groups under German healthcare system. The EVIDEM was the decision support tool we selected to fulfill this MCDA study.

EVIDEM provides a set of generic decision criteria which were made and selected with the goal to support the substantive legitimacy of the decision with regard to the common goal of healthcare system [26]. To highlight the benefits of using EVIDEM: one is to get preferences of which criteria contribute the most to the worth of healthcare interventions in general, which captured as “weights”; the other is to explore the value of the interventions on nodules, which captured by performance scores. Since the EVIDEM provides a set of standardized criteria, this gives the chance for researchers to compare different perspectives of the same criterion on different disease and medical interventions. There are more and more researchers using EVIDEM to explore perspectives on healthcare interventions. Most of them recruited a group of people to make a focus group to do interview face to face [33–36]. Our study used an on-line platform to involve more participants [37], which can avoid being influenced by others from a focus group.

### Perspectives of participants in general on decision criteria

Weights reflect participants’ values and preferences, like what matters most to them. Participants in this study indicated that the most important criteria were ‘Comparative effectiveness’, ‘Type of therapeutic benefit’, ‘Disease severity’, ‘Comparative patients’ perceived health’, ‘Comparative safety’ and ‘Unmet needs’. These results are similar to the results of a survey for the topic of chronic heart disease of all types of stakeholders across the healthcare decision continuum in Germany, which indicated the most important criteria were ‘Clinical effectiveness’, ‘Patients’ perceived health’, ‘Disease severity’, ‘Clinical safety’ and ‘Quality of evidence’ [37].

Compared to physicians, patients tended to assign higher weights to the criteria of patients’ perceived health, higher quality of life for them is much more important after the intervention. This showed also in other study, that patients’ group assigned greater weight to the impact on Health Related Quality of Life [35, 36]. Meanwhile compared to patients, physicians took ‘safety’ more into account. Since physicians are healthcare providers and patients are the receivers of the intervention, physician knows more about what the risk of the intervention, physician indicated keeping the intervention safe and effective were much more important. This difference truly highlights the need for effective communication between physicians and patients, which helps patients to express their need and incorporate their individual priorities in patients-centered healthcare system. Referring to ‘Economic consequence of intervention’, all participants assigned lower values than other criteria, this showed the same in another MCDA study under German healthcare system [37]. These results are in agreement with health professionals, they wish to help patients without focusing on economic constrains [37, 38]. For the patients, they do not have economic constrains in the context of German healthcare insurance system for most diseases [39].

### Appraisal of the intervention on thyroid nodules

Participants used scores to express their views on how each criterion favored for the health intervention based on their own experience and knowledge as well as provided information. Compared to physicians’ group, patients’ group gave higher scores on the current interventions on nodules regarding the therapeutic benefits and preventive benefits. About the prevention benefit, there are different opinions from scientific researches. Although this disease is very common, the causes and risk factors of most nodules and lumps are not clear, it is difficult to prevent this disease [40]. There also one study shows that nutrition can prevent nodules, like Selenium [41]. More evidence and other preventive methods still need to be investigated from more scientific researches.

Referring to ‘Disease severity’, physicians thought nodules as a disease with lower severity than patients’ group. This result is in agreement of many studies, although the prognosis is increasing since the beginning of the twenty-first century, the incidence rates of thyroid cancer and mortality have stabilized in more recent years [42]. Referring to ‘Comparative safety’, the score of physicians’ group was smaller than that of patients’ group, which shows that physicians thought an operation on thyroid gland was not as safe as patients thought. This result is in agreement with other medical researchers, operative complications can be significant, including permanent hypoparathyroidism, vocal fold paralysis, and airway compromise [43]. For ‘Clinical practice guidelines’, the score of physicians’ group was smaller than patients’ group. In principle, as health practitioners, physicians should execute medical interventions based on the recommendation from clinical guideline. The lower score indicates that their appraises of current guideline of thyroid disease should be improved. As it has been observed in practice, the guidelines of interventions on thyroid disease have been updated many times. Like after the revision of 2009 American Thyroid Association (ATA) guideline in 2015,, the rapid increase in thyroid cancer incidence rates has recently slowed, especially among small-sized cancers and women [43]. The appraisal of the interventions on thyroid nodules revealed large differences in performance scores for most criteria. The large variations may come from different perspectives, especially this study has a relative large number of participant compared to other EVIDEM study, this difference has been also observed in the other studies [37, 44].

### Impact of contextual criteria

51% participants considered “Population priorities and access” having a negative impact on the interventions of nodules. This agrees with many studies, that increased incidence of thyroid cancer with attribution to over-diagnosis has been described in most developed countries where patients have high access to health detection. Additional such diagnosis brings a growing amount of thyroid surgery on benign nodules that would not have needed to be removed. Consideration of ‘System capacity and appropriate use of intervention’, 66% participants thought it had a negative impact. This result can also be found other studies, possible changes in exposure to risk factors such as diagnostic radiation overweight, diabetes may increase patients’ medical surveillance, and changes in access to health inspection of thyroid gland may also be the likely explanations [8].

### Limitation of study

The first limitation of this study is the recruitment of participants. Since participants were recruited through public access like medical networks, regional distributors and newspaper announcement, 30 participants from patients’ group (48 participants) are above 60 years old, that might be influence of their perspectives on the interventions. The second limitation of this study is the data analysis methodology. Because the EVIDEM framework introduces a fixed data analysis methodology with mean and SD, large variations have been observed from performance scores for most criteria. Additionally, most data validation methods like normal distribution and standard error of 5% are not suitable for this study. Although this situation also happened to other MCDA studies, especially the ones with EVIDEM Framework, potential uncertainty still existed. The limitation of the fixed criteria should be also taken into consideration, we selected EVIDEM framework with fixed criteria to short time commitment, the applicability of these criteria regarding interventions on thyroid nodules has not been evaluated by other studies, this limitation should be taken into account for further research. Although a definition of all criteria as well as background knowledge was provided to participants in the online questionnaire in German language, the lack of appropriate evidence, difficulties in understanding the complex information may result in lower scores and higher SDs. The same situation regarding contextual criteria should be also taken into consideration, the different understanding of positive impact, neutral impact and negative impact may result in different choice. The good point is that this qualitative survey part just a support tool to help researchers to understand participants’ perspective, which has not been accounted to the quantitative result.

## Conclusion

Our study focused on the current interventions on thyroid nodules, since the diagnose of nodules through routine ultrasound imaging is often the trigger for cascade effects leading to unnecessary follow-up highly discussed by many research teams in many countries. To explore perspectives of the current interventions on thyroid nodules, our study shows physicians and participants’ preferences on each criterion, i.e., they thought unmet needs and disease severity are important criteria, but they gave less scores for both criteria regarding the interventions on thyroid noodles. Especially physicians’ group gave less score for disease severity than patients’ group. Physicians indicated keeping the interventions safe and effective were much more important, patients indicated the quality of life after receiving interventions were much more important. This study provides a new perspective to explore preferences and insights from different groups. Additionally, through comparison between physicians and patients, differences have been highlighted in the study, which can make further better communication between physicians and patients. This study provided a suppurative decision making for physicians and policy makers when they conduct researches on thyroid nodules. We hope the result of this study can contribute to improve diagnosis and treatment of this disease, in addition to ensure sustainable and equitable healthcare resources distribution.

## Data Availability

The datasets used and analyzed during the current study are available from the corresponding author on reasonable request.

## Abbreviations

ACE: Adverse Cascade Effects
ATA: American Thyroid Association
BMBF: The Germany Federal Ministry for Education and Research
EVIDEM: EVIdence based Decision-Making
FAU: Friedrich-Alexander University Erlangen-Nürnberg
MCDA: Multi Criteria Decision Analysis
PRO PRICARE: Preventing Overdiagnosis in Primary Care
QoL: Quality of Life
SD: Standard Deviations
VCx: Value Contribution
